# Mechanical Response and Failure Characteristics of Granite Under In Situ High-Temperature and High-Pressure True Triaxial Conditions

**DOI:** 10.3390/ma19071396

**Published:** 2026-03-31

**Authors:** Yingwen Ma, Xing Fu, Yi Wu, Changsuo Zhou, Jiawen Xue, Xun Zhang, Jin Yang, Shouding Li, Xiao Li, Tianqiao Mao, Yanzhi Hu, Yiming Diao, Xiukuo Sun

**Affiliations:** 1 CNOOC Research Institute Co., Ltd., Beijing 100028, China; mayw@cnooc.com.cn (Y.M.); fuxing@cnooc.com.cn (X.F.); wuyi11@cnooc.com.cn (Y.W.); zhouchs@cnooc.com.cn (C.Z.); xuejw2@cnooc.com.cn (J.X.); 2 College of Safety and Ocean Engineering, China University of Petroleum (Beijing), Beijing 102249, China; ex_zhangxun@cnooc.com.cn (X.Z.); yjin@cup.edu.cn (J.Y.); 3 Key Laboratory of Deep Petroleum Intelligent Exploration and Development, Institute of Geology and Geophysics, Chinese Academy of Sciences, Beijing 100029, China; lixiao@mail.iggcas.ac.cn (X.L.); maotianqiao@mail.iggcas.ac.cn (T.M.); huyanzhi@mail.iggcas.ac.cn (Y.H.); diaoyiming22@mails.ucas.ac.cn (Y.D.); sunxiukuo@mail.iggcas.ac.cn (X.S.)

**Keywords:** in situ testing, temperature, intermediate principal stress, minimum principal stress, true triaxial, granite

## Abstract

**Highlights:**

- Study granite’s behavior under coupled 200 °C/200 MPa conditions using self-developed testing systems.
- Quantified effects of temperature, σ_2_, and σ_3_ on strength, failure mode, and 3D crack complexity.
- Revealed deep-formation granite’s failure mechanisms, supporting drilling and fracturing design.

**Abstract:**

The exploitation of deep oil and gas resources faces challenges posed by complex high-temperature and high-pressure environments. Understanding the mechanical behavior of reservoir rocks under such conditions is therefore critical for ensuring engineering safety. Most existing studies utilize reservoir rock samples at ambient temperature or subjected to high-temperature pretreatment for conventional triaxial tests. However, research on the mechanical response under the coupled conditions of real-time high temperature and in situ true triaxial stress remains limited. To address this gap, this study employed a self-developed in situ true triaxial testing system capable of simultaneous high-temperature and high-pressure loading. Systematic in situ true triaxial mechanical tests were conducted on granite under temperatures up to 200 °C and confining pressures up to 200 MPa. After fracturing, the three-dimensional crack morphology was obtained using CT scanning, and quantitative characterized based on the average crack width and fractal dimension, systematically investigating the effects of temperature, intermediate principal stress (σ_2_), and minimum principal stress (σ_3_) on the mechanical parameters and fracture characteristics of granite. In this study, the results indicate that over the temperature range of 25–200 °C, the peak strength and elastic modulus decrease by approximately 11–18% and 15–40%, respectively, while the peak strain increases by 4–20%. The failure mode transitions gradually from a tensile–shear composite fracture at room temperature to a predominantly shear fracture at elevated temperatures. The rock brittleness is reduced, thus the damage zone expands, and macroscopic fractures decrease. Correspondingly, the average fracture width decreases from approximately 0.66 mm to 0.55 mm, and the fractal dimension increases from about 2.28 to 2.38. An increase in σ_2_ leads to a 19–26% increase in peak strength and a 33–75% increase in elastic modulus, while also increasing the average fracture width and decreasing the fractal dimension. Also, the rock brittleness increases, and the failure mode shifts from tensile–shear composite fracture to shear fracture. An increase in σ_3_ results in an approximately 11% increase in peak strength, a 30% increase in peak strain, and a 21% decrease in elastic modulus, accompanied by a decrease in average fracture width and an increase in fractal dimension. This suppresses the formation of a single dominant fracture surface, consequently increasing the complexity of the fracture morphology. This research reveals the mechanical response and failure laws of deep granite under high-temperature and true triaxial conditions, providing important insights for understanding the mechanical properties of deep reservoir rocks and for the design of drilling and fracturing operations.

## 1. Introduction

The growing global energy demand is driving oil and gas exploration and development into deeper and more challenging environments. In deep resource extraction, reservoir rocks are subjected to high-temperature and high-pressure (HTHP) conditions, which significantly alter their mechanical properties. For instance, in regions such as China’s Bohai Bay Basin, deep buried hill oil and gas reservoirs have been discovered with burial depths exceeding 4000 m [1], temperatures reaching 200 °C, and in situ stresses in the range of 90–120 MPa. For such deep reservoirs, issues related to formation stability and rock deformation directly constrain the efficiency of exploration and development, such as drilling and fracturing. Beyond the limitations imposed by tools and operational practices, the core challenge lies in the fundamental change in rock mechanical response—including strength, hardness, creep behavior, and failure modes—induced by the coupled HTHP environment. This shift promotes a transition from purely brittle fragmentation to more ductile or plastic deformation, thereby critically influencing rock–bit interaction, rate of penetration, and drilling hazards.

One of the primary approaches to study mechanical response under HTHP environment involves laboratory simulation [2,3,4]. Apart from that, numerical simulation also serves as an important tool for studying this issue, although the selection of key parameters still relies on relevant experimental data [5]. However, achieving real-time concurrent high temperature and high pressure in the laboratory while simultaneously monitoring stress–strain behavior remains a considerable technical challenge [6]. Consequently, existing studies usually consider only high-temperature or high-pressure environments, or perform true triaxial tests on specimens after high-temperature treatment.

Experimental studies have demonstrated that temperature significantly influences the mechanical properties of granite. Yin et al. [7] investigated the effect of high temperature on the brittleness of granite, reporting that elevated temperatures lead to a marked reduction in its brittleness. Through uniaxial compression tests, Zhang et al. [8] and Ding et al. [9] found that the peak strength of granite decreases significantly above the range of 300–600 °C. Using true triaxial tests, Su et al. [10] also observed that increasing temperature promotes microcrack density within the rock, thereby reducing its load-bearing capacity, which may account for the degradation of mechanical properties. Furthermore, true triaxial experiments by Liu et al. [11] revealed that as the temperature rises from ambient to 240 °C, the strength reduction of granite can exceed 20%, with smoother fracture surfaces and diminished rock brittleness.

In addition to temperature, confining pressure also significantly influences the physical and mechanical properties of rocks. Zhao et al. [12] conducted true triaxial loading tests to investigate the strength and macro-mesoscopic failure characteristics of rocks with increasing burial depth. Their results indicate that rocks exhibit a pronounced brittle-to-ductile transition as stress increases. When temperature is held constant, the triaxial strength and elastic modulus of specimens increase linearly with rising confining pressure [13]. Gao et al. [14] found that high confining pressure can delay crack coalescence, thereby enhancing the peak strength of rocks. Furthermore, true triaxial experimental studies highlight that the effect of the intermediate principal stress (σ_2_) cannot be neglected. As σ_2_ increases, rocks become more prone to unstable deformation and failure [15,16]. Particularly for rocks with anisotropic characteristics, σ_2_ can interact with bedding or structural planes, leading to more complex failure modes [17].

The combined effect of temperature and confining pressure on rock properties is relatively rare. Meng et al. [18] performed true triaxial loading tests on granite, with results indicating that a high temperature intensifies the anisotropic expansion of the rock, while confining pressure inhibits crack opening, leading to a non-linear variation in rock strength. Ma et al. [19] investigated the true triaxial mechanical behavior of granite at elevated temperatures, finding that temperature significantly influences crack initiation stress, damage stress, and peak strength. Based on these findings, they proposed a temperature-dependent Mogi–Coulomb strength criterion. Similarly, Wang et al. [20] established a rock strength criterion applicable under true triaxial conditions that accounts for plastic deformation and hardening. Yang et al. [21] studied the fracture behavior of granite under high-temperature and high-pressure conditions in the presence of supercritical fluid, discovering that increasing temperature reduces the fracture pressure and increases microcrack volume, while increasing stress elevates the fracture pressure. Yin et al. [22] noted that the mechanical behavior of deep granite under high temperature and pressure is closely related to its grain size, with fine-grained granite exhibiting higher strength and deformation resistance. Yang et al. [23] examined the effects of high temperature on the permeability and mechanical behavior of granite, reporting that high temperature promotes the formation of a microcrack network, resulting in a significant increase in permeability—an effect that can be partially suppressed by confining pressure.

A buried hill reservoir refers to a paleo-geomorphic uplift formed by basement rock that becomes a concealed, deeply buried structure after being covered by younger sedimentary strata [24,25]. Characterized by large reservoir thickness, strong heterogeneity in physical properties, and substantial economic reserves [26,27], such reservoirs have become an important type of oil and gas accumulation discovered worldwide [28]. Prominent examples include the Archean buried hill reservoirs in the Bohai Bay Basin of China, such as Bozhong 19-6, Bozhong 13-2, and Bozhong 26-6 oil and gas fields, where formation temperatures can reach up to 200 °C and in situ pressures range from 90 to 120 MPa. The efficient development of these resources relies on a thorough understanding of the physical and mechanical properties of the reservoir rocks. Therefore, research on rock mechanical behavior under corresponding high-temperature and high-pressure conditions is essential.

According to the before-mentioned research, true triaxial tests under in situ real-time HTHP conditions remain relatively scarce, especially with temperatures and confining pressures exceeding 100 °C and 100 MPa, respectively. To address this gap, the primary goal of this study is to systematically investigate the mechanical properties, failure characteristics, and 3D fracture morphology of granite under in situ real-time HTHP conditions. This investigation is carried out using a self-developed HTHP true triaxial rock mechanics testing system. The system enables testing of granite—a rock analogous to the reservoir lithology of the Bohai buried hills—under simulated downhole conditions reaching temperatures of 200 °C and confining pressures of 200 MPa. This research specifically elucidates the individual and combined influences of temperature, σ_2_, and σ_3_ on these characteristics. The findings are expected to provide theoretical support and practical guidance for the safe and efficient development of deep oil and gas reservoirs.

## 2. Materials and Methods

### 2.1. Sample Selection and Preparation

In order to study the mechanical response and failure characteristics of the rocks in the Bohai Bay Basin of China under HTHP conditions, such samples need to be collected. However, due to regional tectonics and geological history, typical outcrops are not found around the basin perimeter. Furthermore, the limited number of deep downhole cores is extremely valuable and often of insufficient size for extensive rock mechanics testing. Consequently, utilizing analogous rocks with a similar mineralogical composition and mechanical properties to the downhole cores has become a practical alternative, so the field investigations on outcrop samples from Xintai City, Shandong Province, China, was conducted. After comparative analysis of various rock types, the Tai-2 rock (a gray–black granodiorite) was selected as the test sample for this study.

**Figure 1:**
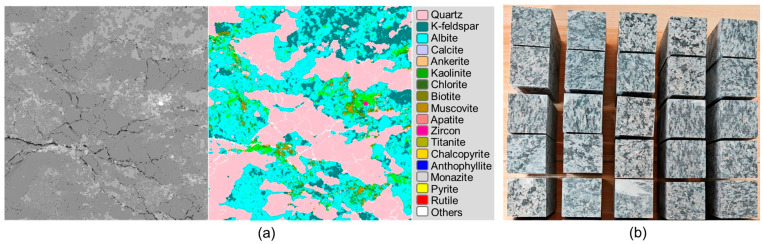
Granite samples for the high-temperature and high-pressure true triaxial test: (**a**) mineral composition; (**b**) tested specimens.

The results of automated mineralogical analysis via scanning electron microscopy for the Tai-2 rock are shown in Figure 1a. Its mineral composition, compared with that of the Archean buried hill reservoir rock from the Bozhong area, is presented in Table 1. Furthermore, P-wave velocity measurements and uniaxial compression tests were conducted on both the Tai-2 rock and reservoir core, and the comparative results are listed in Table 2. It can be observed that the mineral composition and physical–mechanical properties of the Tai-2 granodiorite closely match those of the actual reservoir core. Therefore, Tai-2 granodiorite was selected in this study as a representative analog for investigating the mechanical behavior of the Archean buried hill reservoir rock. It should be noted that while this study employs a granite analog rather than the actual deep reservoir core, the strong similarity in mineral composition and mechanical properties indicates that, in terms of mechanical parameters and failure mechanisms, the experimental results remain highly relevant and provide valuable insights for the target buried hill reservoir.

The selected Tai-2 granodiorite blocks were machined into cuboid specimens measuring 40 mm × 40 mm × 80 mm using wire-electrical discharge machining cutting. Dimensional accuracy was controlled within ±0.3 mm, and the end face flatness error was maintained below 0.05 mm to ensure uniform stress distribution during testing and to minimize result deviations caused by specimen surface imperfections (Figure 1b). After machining, the P-wave and S-wave velocities of all prepared specimens were measured to ensure sample consistency. The measurements were conducted using a non-metallic ultrasonic testing analyzer (see Figure S1 in the Supplementary Materials) with a time-measurement accuracy of 0.4 µs. To ensure optimal acoustic coupling between the transducers and the rock, a thin layer of petroleum jelly was applied to both ends of the specimen. Specimens exhibiting significant deviations in wave velocity were excluded from the test series. The measured wave velocities for all specimens are listed in Table S1 in the Supplementary Materials. A total of 25 test specimens meeting these criteria were prepared.

### 2.2. Experimental Equipment

**Figure 2:**
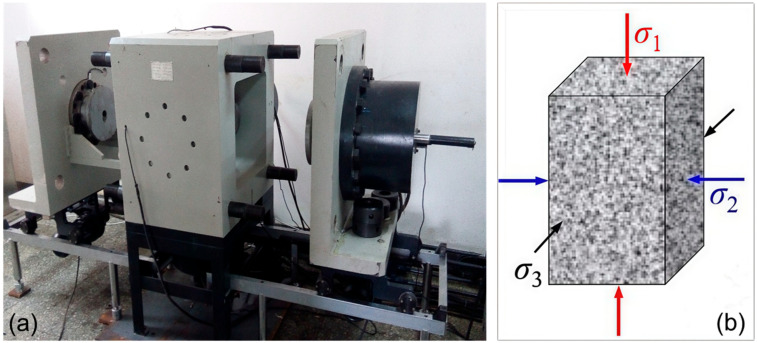
(**a**) High-temperature and high-pressure true triaxial rock mechanics experimental system; (**b**) schematic of true triaxial loading directions.

This study employed a high-temperature and high-pressure true triaxial rock mechanics testing system, developed by the Institute of Geology and Geophysics, Chinese Academy of Sciences, Beijing, China (Figure 2a). This apparatus can simulate complex deep subsurface environments, with a maximum temperature of 240 °C and a maximum confining pressure of 240 MPa. The sample was heated from all four lateral sides to ensure a uniform temperature distribution, with a temperature measurement accuracy of ±0.5 °C. The system was equipped with six independent sets of Linear Variable Differential Transformer (LVDT) displacement sensors arranged around the fixture. These sensors enabled independent, high-precision measurements of the three-dimensional deformation of the specimen within a range of 0–5 mm.

The confining pressures (σ_2_ and σ_3_) and the axial pressure (σ_1_) in the self-developed HTHP true triaxial system were independently applied and controlled by three separate, high-precision electro-hydraulic servo systems. Each loading direction was equipped with a servo valve and a high-accuracy pressure transducer (with an accuracy of ±0.1% FS). This setup enabled a closed-loop, servo-controlled loading process. The pressure was generated by a hydraulic power unit and transmitted to the specimen via the rigid loading platens. During the experiment, the system automatically regulated the servo valves to achieve the specified stress increase rates and maintains the confining pressures (σ_2_ and σ_3_) at their target values with high stability once they reached the set points, ensuring a constant stress boundary condition during the subsequent axial (σ_1_) loading to failure.

To ensure a uniform temperature distribution within the granite specimen during the true triaxial tests, a dedicated heating system was employed. The sample was heated from all four lateral sides via integrated heating elements within the true triaxial loading plates. The assembly was externally wrapped with thermal insulation pads to minimize heat loss to the pressure vessel and the environment. Prior to the formal mechanical tests, calibration experiments were conducted to verify temperature uniformity. This was done by drilling a small hole at the center of a representative sample, inserting a temperature sensor, and monitoring the thermal response. The results confirm that the temperature at the center of the rock specimen reached the target value (with a deviation of less than ±2 °C) after approximately 40 min of heating. Therefore, in all subsequent mechanical tests, the specimen was heated to the target temperature and then held for a stabilization period of one hour before mechanical loading commenced, ensuring a thoroughly uniform temperature field throughout the sample volume.

To analyze the internal fracture networks, a high-energy accelerator CT experimental system developed by the Institute of Geology and Geophysics, Chinese Academy of Sciences, Beijing, China was employed. This system can achieve a maximum energy value of 6 MeV, which is sufficient to penetrate rock samples with side lengths up to 300 mm. Following the high-temperature and high-pressure true triaxial mechanical tests, each failed specimen was scanned using this system to obtain detailed images of its internal fracture structure, so the influence of temperature, σ_2_, and σ_3_ on fracture morphology could be investigated. The resolution of the CT images obtained in this experiment was 0.129 mm per pixel.

### 2.3. Experimental Method

The experimental scheme was designed based on actual downhole temperature and pressure measurements from a well in the Bohai Basin at different depths (4000 m, 5000 m, and 5700 m), as detailed in Table 3. A series of high-temperature and high-pressure true triaxial compression tests were conducted accordingly. The tests systematically applied different temperatures, σ_3_, and σ_2_ to the specimens. To investigate the influence of temperature variation on the mechanical properties and failure characteristics in detail, except for the original temperatures corresponding to each target depth, tests at temperatures of 25 °C, 50 °C, and 100 °C were also performed. Similarly, at the respective in situ formation temperatures, the effects of σ_3_ and σ_2_ were studied by independently varying these stress parameters. The specific experimental parameters and grouping are listed in Table 4.

Before testing, the specimen was first installed in the true triaxial fixture. Then the prepared sample was placed into the pressure vessel, and the true triaxial loading assembly was sealed. The temperature loading system was then activated to heat the specimen to the target temperature at a controlled rate. At first, the three principal stresses (σ_1_, σ_2_, σ_3_) were simultaneously increased at a rate of 0.2 MPa/s. The orientations of these three principal stresses acting on the cuboid specimen are schematically illustrated in Figure 2b. Once the σ_3_ reached its predetermined value, it was held constant. The σ_2_ and maximum principal stress (σ_1_) were then continually increased until the target value of σ_2_ was reached, then σ_2_ was also maintained. Finally, σ_1_ was increased under servo-controlled displacement loading until specimen failure occurred. This means that, during the main loading phase leading to failure, σ_2_ and σ_3_ were maintained constant, while σ_1_ was monotonically increased. Throughout the entire loading process, the loads and deformations in the three principal directions were continuously recorded to obtain the complete stress-strain evolution. After failure, the specimen was carefully extracted and subjected to CT scanning to characterize its internal fracture morphology.

### 2.4. Data Processing and Calculation of Mechanical Parameters

The key mechanical parameters reported in this study—peak strength, peak strain, elastic modulus, and Poisson’s ratio—were derived from the recorded stress–strain curves according to the following standards to ensure consistency and reproducibility:

Peak strength: The peak triaxial compressive strength was taken as the maximum value of the axial stress (σ_1_) recorded on the stress–strain curve prior to specimen failure.

Peak strain: The peak strain corresponds to the axial strain value at which the peak strength occurs on the stress–strain curve.

Elastic modulus: The static elastic modulus was calculated as the slope of the most linear portion of the axial stress (σ_1_) versus axial strain (ε_1_) curve during the loading phase. To obtain a representative and consistent value, the linear regression was performed over the stress interval from about 30% to 70% of the peak strength, thereby avoiding the initial non-linear compaction region and the non-linear segment near the peak.

Poisson’s ratio: Poisson’s ratio was determined for the same linear elastic region used for the calculation of E. It was computed as the negative ratio of the average lateral strain to the axial strain. The average lateral strain was calculated from the two independent lateral strain measurements (in the directions of σ_2_ and σ_3_).

### 2.5. Quantitative Evaluation of Fracture Complexity

After sample failure, the three-dimensional structure of the fractured rock was obtained through CT scanning and subsequent 3D reconstruction of the image slices. The fracture networks within the sample were extracted using a combination of threshold segmentation and manual post-processing tools implemented in the commercial CT data analysis software VG Studio Max (Version 3.2). To quantitatively characterize the complexity of the fractures and thereby systematically analyze the influence of temperature, σ_2_, and σ_3_ on the fracturing process, two metrics were calculated: the average fracture width and the fractal dimension (D).

The average fracture width in samples was determined using the built-in local thickness analysis module in the software VG Studio Max (Version 3.2), which implements the “maximum inscribed sphere” or “sphere-fitting” method. This algorithm calculates, for each voxel within the extracted 3D fracture volume, the diameter of the largest sphere that can be inscribed within the fracture while containing that voxel. This diameter represents the local fracture aperture. The “average fracture width” reported in this study is the mean value of all these local aperture values across the entire fracture network, providing an objective measure that accounts for multiple failure planes and spatial variations.

The fractal dimension was determined using the Box-Counting Method. The principle of this method is as follows: the 3D fracture network is covered with cubic grids of varying side lengths (ε). For each grid size ε, the minimum number of boxes, N(ε), required to cover all parts of the fracture network is counted. According to fractal theory, for a scale-invariant structure, N(ε) and ε satisfy the power law relationship given in Equation (1). Taking the natural logarithm of both sides of this equation yields the linear form shown in Equation (2):

N(ε) ∝ ε^(-D)(1)

log[N(ε)] = D·log(1/ε) + C (2)

Performing least-squares linear regression on the data points of log[N(ε)] versus log(1/ε) in a logarithmic coordinate system yields a fitted line whose slope provides an estimate of the fractal dimension D. This parameter quantitatively describes the spatial complexity and irregularity of the fracture network: a D value closer to 2 indicates that the fractures tend to approximate simpler, more planar two-dimensional features. Conversely, a D value closer to 3 reflects fractures with high degrees of tortuosity, branching, and three-dimensional spatial propagation, thus representing greater complexity.

## 3. Results

### 3.1. Results of True Triaxial Tests Under Different Temperature Conditions

**Figure 3:**
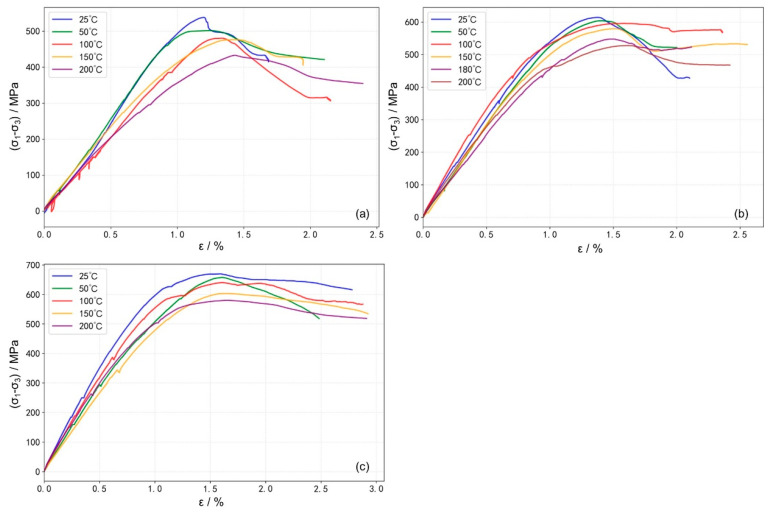
Stress–strain curves under different temperature conditions: (**a**) stress conditions at 4000 m depth: σ_3_ = 64 MPa, σ_2_ = 80 MPa; (**b**) stress conditions at 5000 m depth: σ_3_ = 80 MPa, σ_2_ = 100 MPa; (**c**) stress conditions at 5700 m depth: σ_3_ = 96 MPa, σ_2_ = 120 MPa.

Stress–strain curves of granite under different temperature conditions (25–200 °C) were obtained from the HTHP true triaxial compression experiments, as shown in Figure 3. Under the confining pressure corresponding to a depth of 4000 m (Figure 3a), the stress–strain curves exhibit typical elastic stages followed by post-peak strain-softening behavior. During the elastic stage, stress and strain maintain an approximately linear relationship, indicating negligible irreversible deformation or cracking. After reaching the peak strength, the specimens undergo rapid strain softening, characterized by a sharp stress drop and a rapid increase in strain, which signifies a swift loss of load-bearing capacity. With increasing temperature, the slope of the linear elastic phase decreases slightly, and the curvature near the peak strength becomes more gradual. The failure process is prolonged compared to that at room temperature, suggesting a reduction in rock brittleness and the emergence of plastic deformation characteristics.

**Figure 4:**
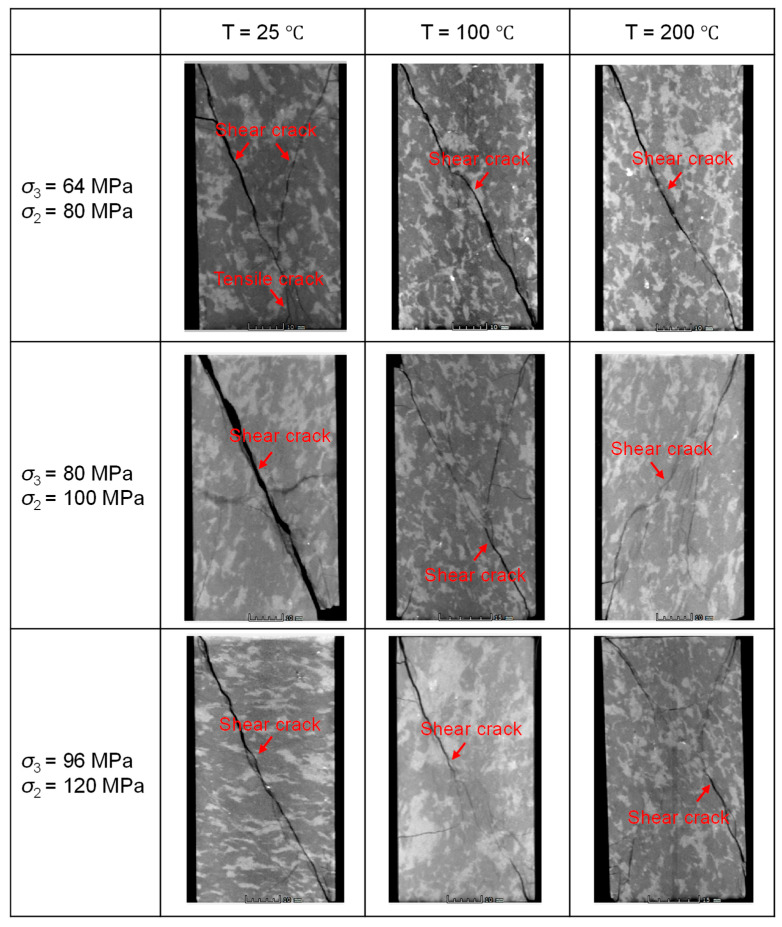
CT slices of granite after failure under different temperature conditions (The sample in the CT slice is 40 cm wide and 80 cm high).

After specimen failure, the internal fracture distribution was obtained through CT scanning. Figure 4 presents images along the direction of σ_1_ for each specimen under different temperature and in situ stress conditions, where the black regions in samples represent cracks. The three rows of CT images, from top to bottom, correspond to the stress conditions (with σ_3_ and σ_2_ progressively increasing) associated with burial depths of 4000 m, 5000 m, and 5700 m, respectively. The three columns, from left to right, represent test temperatures of 25 °C, 100 °C, and 200 °C, respectively. As shown in the figure, under identical σ_3_ and σ_2_ conditions, an increase in temperature from 25 °C to 200 °C leads to a gradual decrease in fracture width, more complex fracture morphology, and a transition in failure mode from a combined tensile–shear fracture to a more distributed damage pattern. At a constant temperature, a simultaneous increase in σ_3_ and σ_2_ also results in a progressive reduction in fracture width.

When the experimental confining pressures (σ_3_ and σ_2_) correspond to the in situ conditions at a 5000 m depth (Figure 3b), it is evident that, compared to the 4000 m condition (Figure 3a), the stress–strain curves at all temperatures exhibit a more flattened trend near peak stress. This indicates a gradual enhancement in sample ductility with increasing confining pressure. Notably, under these stress conditions (σ_3_ = 80 MPa, σ_2_ = 100 MPa), the curves show a tendency for stress to increase or stabilize in the post-peak stage. This suggests that, after initial failure, under the constraint of the horizontal principal stresses, the internal structure undergoes rearrangement, the rock can re-form a relatively stable load-bearing architecture. At lower temperatures, distinct macroscopic fractures develop, accompanied by surrounding secondary cracks. As temperature increases, the failure mode gradually transitions to a more distributed damage pattern, macroscopic fractures become less distinct, rock brittleness weakens, and ductility is enhanced (Figure 4).

When the experimental confining pressure is increased to match the in situ conditions at a 5700 m depth (Figure 3c), the post-peak stress declines very slowly with increasing strain. This indicates a further enhancement in sample ductility and a marked transition from brittle to ductile behavior. Unlike the response observed under the 5000 m condition (Figure 3b), the post-peak stress under this higher confinement shows no tendency to increase within the measured strain range for any tested temperature. At this confined stress level, brittle fracture is significantly suppressed. At lower temperatures, distinct macroscopic fractures are still observable; however, as temperature increases, the failure mode transitions to a damage-dominated pattern, and macroscopic fractures become less distinct (Figure 4).

### 3.2. Results of True Triaxial Tests Under Different σ_2_ Conditions

**Figure 5:**
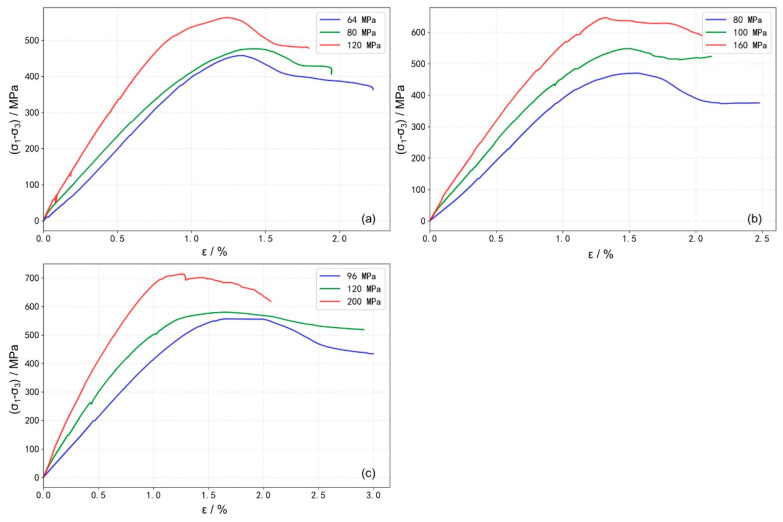
Stress–strain curves under different σ_2_ conditions: (**a**) temperature and pressure conditions at 4000 m depth: σ_3_ = 64 MPa, T = 150 °C; (**b**) temperature and pressure conditions at 5000 m depth: σ_3_ = 80 MPa, T = 180 °C; (**c**) temperature and pressure conditions at 5700 m depth: σ_3_ = 96 MPa, T = 200 °C.

**Figure 6:**
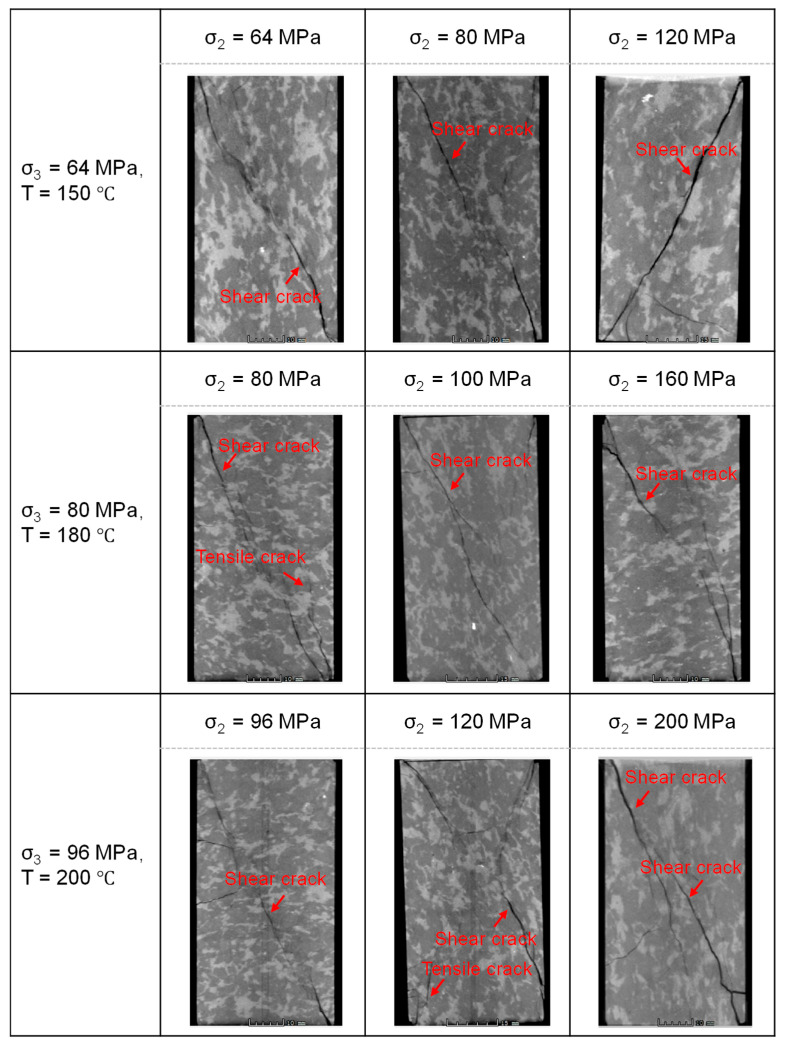
CT slices of granite after failure under different σ_2_ conditions (The sample in the CT slice is 40 cm wide and 80 cm high).

The stress–strain curves of granite under varying σ_2_ conditions are presented in Figure 5. With constant σ_3_ and temperature, an increase in σ_2_ leads to notable changes in the curve morphology: the slope of the elastic stage increases, and the post-peak stress drop occurs slightly more rapidly. Figure 6 displays CT images of the failed granite specimens under different σ_2_ conditions. Observations from each row in Figure 6 show that as σ_2_ increases, the fracture width within the specimens increases, the fracture mode changes, tensile fractures diminish gradually while shear fractures become more prominent, and the rock exhibits slightly enhanced brittle failure characteristics. These results indicate that σ_2_ significantly influences the mechanical behavior and failure mode of the rock.

### 3.3. Results of True Triaxial Tests Under Different σ_3_ Conditions

**Figure 7:**
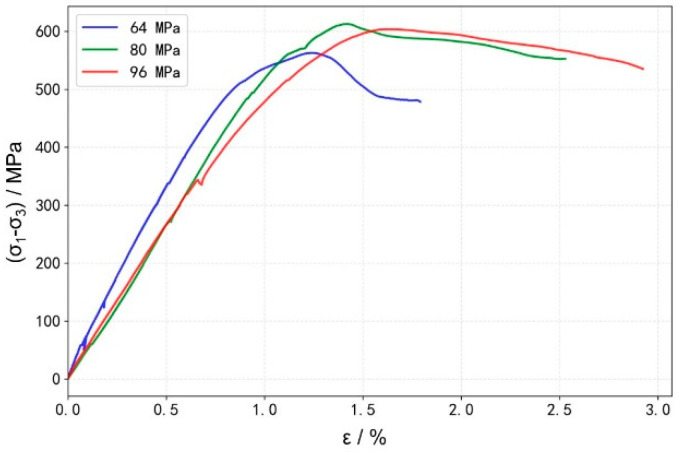
Stress–strain curves under different σ_3_ conditions: σ_2_ = 120 MPa, T = 150 °C.

**Figure 8:**
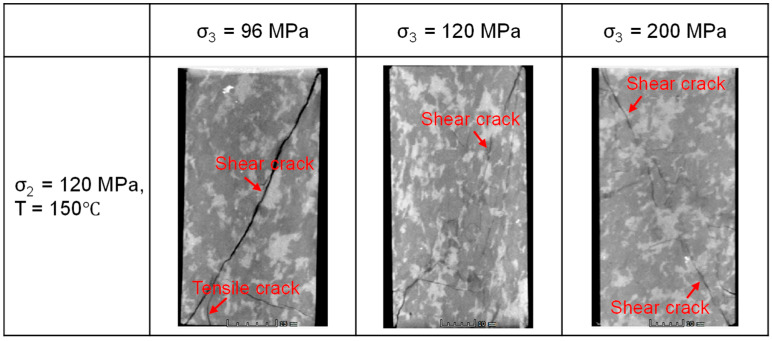
CT slices of granite after failure under different σ_3_ conditions (The sample in the CT slice is 40 cm wide and 80 cm high).

Figure 7 presents the stress–strain curves of granite under varying σ_3_ conditions. Overall, under constant σ_2_ and temperature, an increase in σ_3_ induces distinct changes in the mechanical response: the slope of the elastic stage decreases, the post-peak stress drops more slowly, and the failure mode shifts, with an increase in small fractures distributed among the sample. Correspondingly, the rock’s failure behavior changes from brittle to more ductile characteristics, as corroborated by the fracture morphologies shown in Figure 8, where the cracks become thinner and more numerous. These results demonstrate that the σ_3_ also exerts a significant influence on the mechanical behavior of granite under true triaxial conditions. A comprehensive summary of the key mechanical parameters obtained under all tested conditions is provided in Table 5.

## 4. Discussion

### 4.1. Influence of Temperature on Mechanical and Fracture Characteristics of Granite Under True Triaxial Condition

#### 4.1.1. Variation of Mechanical Parameters with Temperature

**Figure 9:**
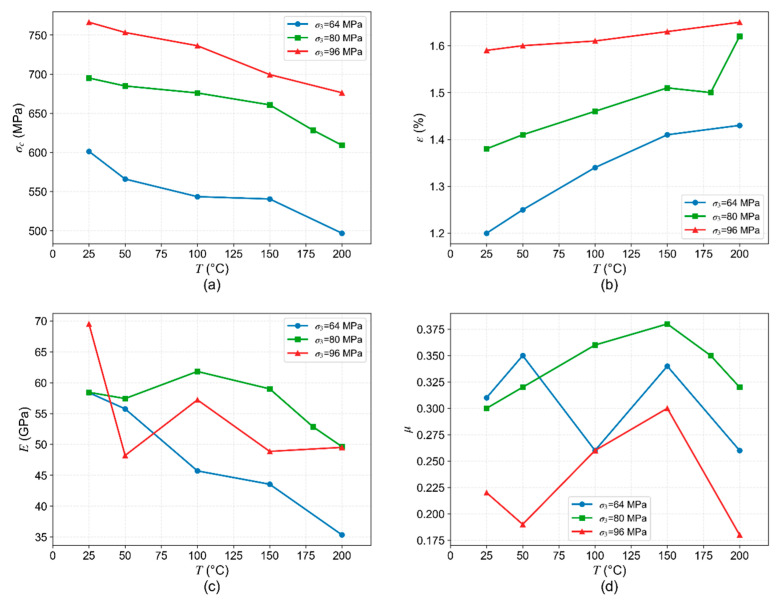
Variation of mechanical parameters with temperature: (**a**) peak strength (σc); (**b**) peak strain (ε); (**c**) elastic modulus (E); (**d**) Poisson’s ratio (μ).

When the confining pressure is held constant, variations in temperature significantly influence the mechanical parameters of the rock. This is attributed to the non-uniform thermal expansion of mineral grains within the rock as temperature rises, which induces fractures both at grain boundaries and within the grains [29]. Thus, as the temperature increases from 25 °C to 200 °C, microcracks generate and reduce the rock’s load-bearing capacity, as well as enhance its deformability, resulting in a gradual decrease in the triaxial compressive strength and an increase in the peak strain (Figure 9a,b). At room temperature, the rock primarily undergoes elastic deformation with minimal plastic strain before peak strength, and a relatively small peak strain. With increasing temperature, the rock’s brittleness decreases and its ductility enhances, allowing for greater plastic deformation during loading and thus leading to an increase in peak strain.

As the temperature increases from 25 °C to 200 °C, the elastic modulus generally exhibits a decreasing trend, as shown in Figure 9c. Under lower confining pressure (σ_3_ = 64 MPa), the elastic modulus decreases monotonically with rising temperature, indicating that the rock deformability is enhanced. However, under high confining pressures (σ_3_ = 80 MPa, σ_3_ = 96 MPa), pores and microcracks within the rock are compressed, resulting in a denser structure and enhanced interactions. This effect partially mitigates the influence of temperature on rock deformation. Concurrently, due to the variability among samples, the elastic modulus no longer decreases monotonically under high confinement, though it still displays an overall declining trend.

In contrast, Poisson’s ratio exhibits no clear trend within the temperature range of 25–200 °C, with variations generally confined within ±0.1, as shown in Figure 9d. Poisson’s ratio, which reflects the ratio of lateral to axial strain, indicates that temperature has no significant effect on this ratio. Similarly, under different in situ stress conditions, Poisson’s ratio also shows no systematic change. This suggests that Poisson’s ratio is a relatively stable parameter, less sensitive to variations in both temperature and confining pressure. Therefore, in the mechanical analysis for deep oil and gas extraction engineering in this study area, it can be treated as a stable material constant.

Quantitatively, as shown in Figure 9, over the investigated temperature range of 25–200 °C, the peak strength and elastic modulus of the granite decrease by approximately 11–18% and 15–40%, respectively, while the peak strain increases by 4–20%. These data systematically confirm the material softening and enhanced ductility induced by elevated temperature.

#### 4.1.2. Variation of Fracture Morphology with Temperature

**Figure 10:**
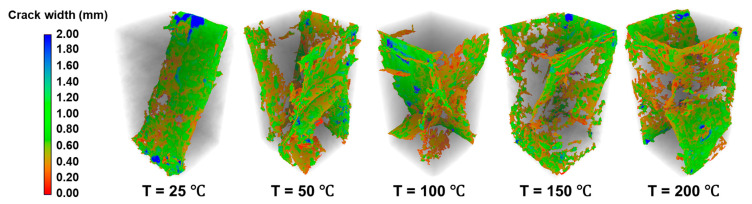
Influence of temperature on fracture morphology (stress conditions at 5700 m depth: σ_3_ = 96 MPa, σ_2_ = 120 MPa).

**Figure 11:**
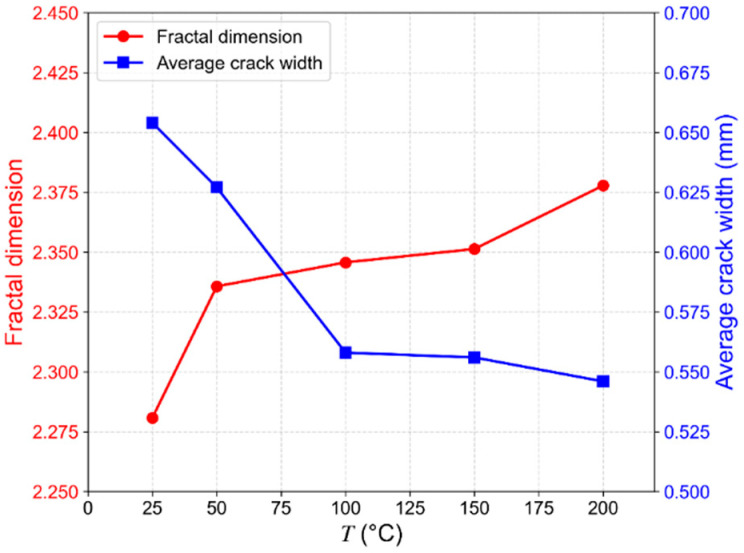
Variation in fracture fractal dimension and average width with temperature (stress conditions at 5700 m depth: σ_3_ = 96 MPa, σ_2_ = 120 MPa).

Through three-dimensional reconstruction and fracture extraction from CT slices of rock specimens tested at different temperatures, the evolution of rock fracture morphology can be clearly observed. Taking the in situ stress conditions at 5700 m depth as an example (σ_3_ = 96 MPa, σ_2_ = 120 MPa; Figure 10), as the temperature increases from 25 °C to 200 °C, the rock failure mode transforms from a relatively brittle fracture pattern characterized by distinct macroscopic fractures, to a damage-dominated failure mode, where macroscopic fractures become less distinct and the surrounding damage zone expands. This transition reflects a reduction in rock brittleness and an enhancement of ductility. The variations in fractal dimension and average width of cracks with temperature are shown in Figure 11. The average fracture width decreases from 0.655 mm to 0.546 mm, while the fractal dimension increases from 2.281 to 2.378, indicating an increase in fracture complexity. This behavior is attributed to the non-uniform and anisotropic thermal expansion of constituent minerals. Under high-temperature conditions, non-uniform thermal stresses develop within the granite, inducing discontinuous deformation. This promotes the widespread activation, propagation, and interconnection of microcracks, ultimately forming a more complex fracture network.

### 4.2. Influence of σ_2_ on Mechanical and Fracture Characteristics of Granite Under True Triaxial Condition

#### 4.2.1. Variation in Mechanical Parameters with σ_2_

**Figure 12:**
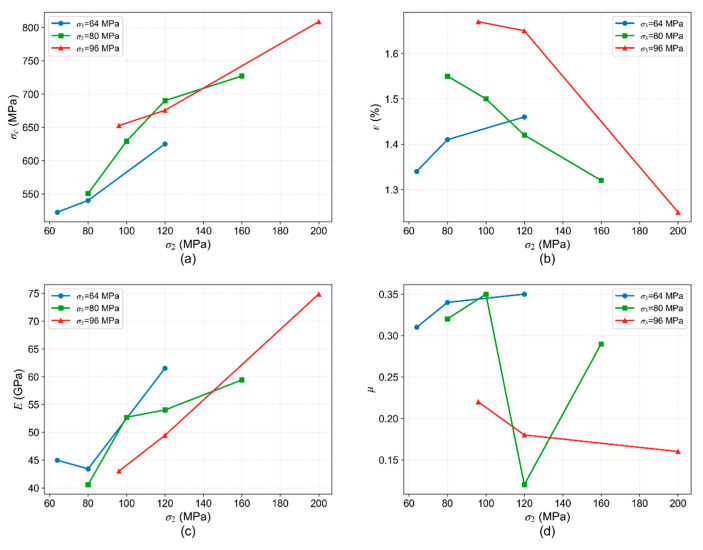
Variation in mechanical parameters with σ_2_: (**a**) peak strength (σ_c_); (**b**) peak strain (ε); (**c**) elastic modulus (E); (**d**) Poisson’s ratio (μ).

At the investigated depths of 4000 m, 5000 m, and 5700 m, the original in situ formation temperatures are 150 °C, 180 °C, and 200 °C, and the σ_3_ are 64 MPa, 80 MPa, and 96 MPa, respectively. Variations in σ_2_ under these conditions lead to distinct changes in mechanical parameters, as summarized in Figure 12. The influence of σ_2_ on strength parameters, particularly peak strength and peak strain, is pronounced (Figure 12a,b). At the shallower depth (4000 m), an increase in σ_2_ from 64 MPa to 120 MPa results in higher peak strength and greater peak strain. However, at greater depths (5000 m and 5700 m), although the triaxial compressive strength also increases with σ_2_, the peak strain decreases. This indicates that under higher temperature and confinement, an increase in the σ_2_ enhances rock brittleness, leading to failure at progressively smaller deformations.

At different depths, the elastic modulus generally increases with rising σ_2_ (Figure 12c), which is likely attributable to the closer contact of grains with each other, enhancing intergranular friction and interlocking, thereby improving the rock’s resistance to deformation. However, the Poisson’s ratio fluctuates within a relatively narrow range under different σ_2_ conditions, not exhibiting a clear increasing or decreasing trend (Figure 12d).

In summary, the quantitative analysis presented in Figure 12 indicates that an increase in σ_2_ leads to a 19–26% enhancement in peak strength and a 33–75% increase in elastic modulus.

#### 4.2.2. Variation in Fracture Morphology with σ_2_

**Figure 13:**
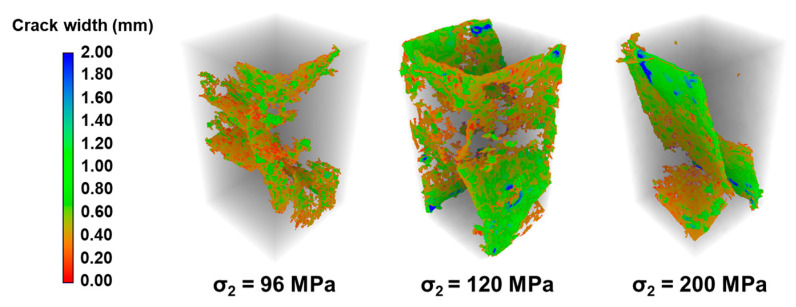
Influence of σ_2_ on fracture morphology (temperature and pressure conditions at 5700 m depth: σ_3_ = 96 MPa, T = 200 °C).

**Figure 14:**
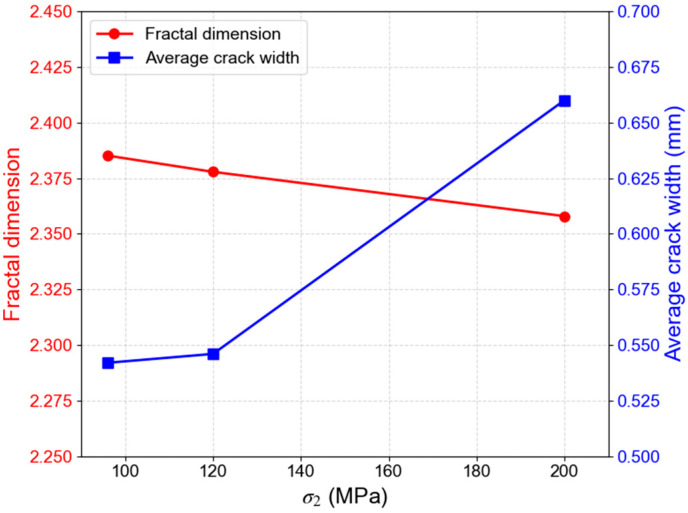
Variation in fracture fractal dimension and average width with σ_2_ (temperature and pressure conditions at 5700 m depth: σ_3_ = 96 MPa, T = 200 °C).

The influence of σ_2_ on rock fracture morphology is also significant, as illustrated in Figure 13. Under constant temperature and σ_3_ (σ_3_ = 96 MPa, T = 200 °C), as σ_2_ increases from 96 MPa to 200 MPa, the fracture characteristics gradually change. When σ_2_ equals σ_3_ (σ_2_ = σ_3_ = 96 MPa), the main fracture is less prominent, and the failure presents a damage-dominated state exhibiting the highest ductility. With increasing σ_2_, the failure mode progressively changes to tensile–shear composite failure, and finally to shear-dominated failure. The corresponding variations in the fracture fractal dimension and average width with σ_2_ are shown in Figure 14. The average fracture width increases from 0.542 mm to 0.660 mm, and the fractal dimension decreases from 2.398 to 2.345. These trends indicate that a higher σ_2_ imposes greater confinement on the rock, altering crack propagation from a multi-branching pattern to a more localized shear-slip mode. This enhanced confinement suppresses the initiation and branching of secondary cracks, resulting in a final fracture morphology that is simpler and more regular.

### 4.3. Influence of σ_3_ on Mechanical and Fracture Characteristics of Granite Under the True Triaxial Condition

#### 4.3.1. Variation in Mechanical Parameters with σ_3_

**Figure 15:**
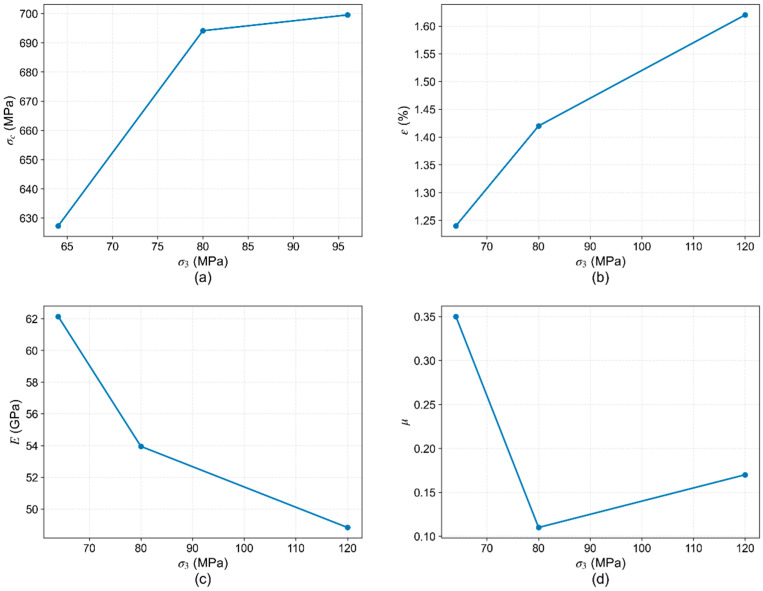
Variation in mechanical parameters with σ_3_: (**a**) peak strength (σc); (**b**) peak strain (ε); (**c**) elastic modulus (E); (**d**) Poisson’s ratio (μ).

In this study, with the temperature and σ_2_ held constant at 150 °C and 120 MPa, the influence of the σ_3_ was examined. As it increases from 64 MPa to 96 MPa, the peak strength shows a moderate increase, accompanied by an increase in peak strain. Conversely, the elastic modulus decreases with increasing σ_3_. Poisson’s ratio, however, it continues to show no consistent trend (Figure 15).

The consolidated data in Figure 15 show that an increase in σ_3_ results in an approximately 11% increase in peak strength, a 30% increase in peak strain, and a 21% decrease in elastic modulus.

#### 4.3.2. Variation in Fracture Morphology with σ_3_

**Figure 16:**
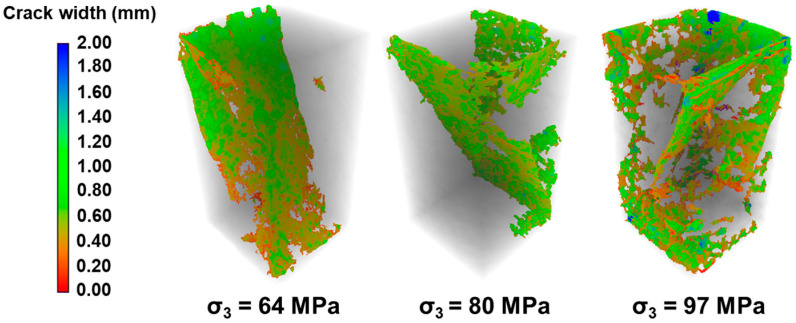
Influence of σ_3_ on fracture morphology (σ_2_ = 120 MPa, T = 150 °C).

**Figure 17:**
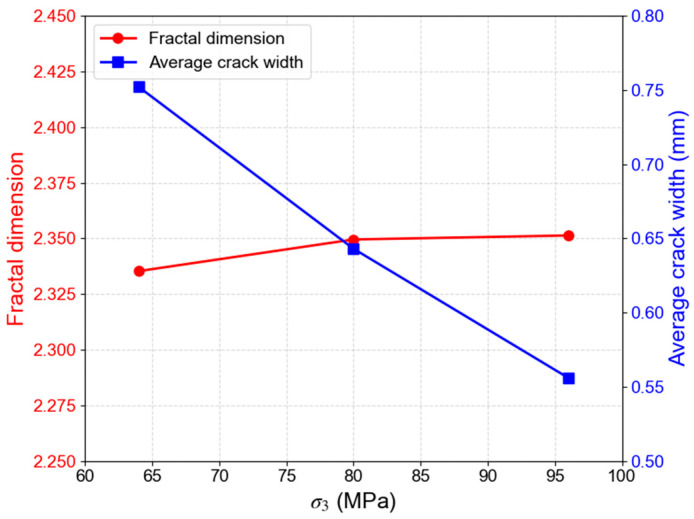
Variation in fracture fractal dimension and average width with σ_3_ (σ_2_ = 120 MPa, T = 150 °C).

Under constant σ_2_ and temperature (σ_2_ = 120 MPa, T = 150 °C), an increase in σ_3_ from 64 MPa to 96 MPa leads to an observable transition in failure mode, from predominantly shear failure to a shear-damage failure mode (Figure 16). This shift is accompanied by enhanced sample ductility. The variations in the fracture fractal dimension and average width with σ_3_ are shown in Figure 17. The average fracture width decreases from 0.752 mm to 0.556 mm, and the fractal dimension grows from 2.351 to 2.385. This indicates that a higher σ_3_ inhibits the formation of a single, dominant fracture plane, promoting the branching and interconnection of fractures along multiple pathways, thereby enhancing the complexity of the fracture network.

### 4.4. Deformation and Failure Mechanism

A comprehensive analysis of the aforementioned HTHP true triaxial test results indicates that the deformation and failure of granite under such conditions are governed by the combined effects of temperature and the true triaxial stress state. Temperature primarily influences the overall mechanical response of the rock and the dispersion of fracture development. In addition, the three-dimensional stress state, through the different constraining effects of σ_2_ and σ_3_, controls the transition of the failure mode among tensile, shear, and damage mechanisms, as well as their composite forms.

With increasing temperature, the specimens are characterized by reduced strength and elastic modulus alongside increased peak strain, reflecting overall material softening and enhanced ductility. In terms of fracture morphology, growing temperatures lead to finer and more complex fractures: as the temperature rises from 25 °C to 200 °C, the average fracture width decreases while the fractal dimension increases. This indicates that failure is no longer governed by a few dominant macroscopic fractures, but is instead achieved through a more numerous, finer, and spatially dispersed fracture network, culminating in distributed damage and macroscopic instability.

Under a true triaxial stress state, σ_2_ and σ_3_ exhibit opposing controlling effects on the fracture mode. When the temperature and σ_3_ remain constant, an increase in σ_2_ leads to a reduction in tensile fractures and an increase in shear fractures. The failure mode progressively shifts from tension–shear composite failure to shear failure. Concurrently, the average fracture width increases, and the fractal dimension decreases, meaning that the fracture morphology tends to become simpler. This indicates that stronger σ_2_ confining constraints inhibit the initiation and branching of fractures, causing failure to localize. Conversely, when temperature and σ_2_ are held constant, an increase in σ_3_ results in more small fractures distributed among the rock. The failure transforms from a shear or composite type towards a damage mode, with decreased fracture width and an increased fractal dimension, suggesting a greater propensity for forming complex fracture networks within the rock.

Therefore, the combined influence of temperature, σ_2_, and σ_3_ on the fracture mechanism can be summarized as follows: increased temperature promotes enhanced rock ductility and greater complexity of the damage zone; an increase in σ_2_ drives the failure towards a mode with an increased shear component, while inhibiting crack opening and branching, thereby reducing the complexity of the fracture network; and an increase in σ_3_ suppresses the formation of a single dominant fracture surface, consequently increasing the complexity of the fracture morphology.

For drilling engineering, such a pattern implies that, during operations in deep HTHP buried hill formations, both rock fragmentation and wellbore stability are governed by the combined effects of temperature and the three-dimensional in situ stress state. The reduced load-bearing capacity and the development of finer, more dispersed fractures in high-temperature environments may influence rock-breaking efficiency and the mechanical response of the formation. Meanwhile, variations in σ_2_ and σ_3_ will further control whether rock failure tends towards complex or localized bands, thereby directly affecting wellbore instability risk and the operation for drilling parameters. Consequently, in the design of deep wells, both thermal effects and the true triaxial in situ stress state should be incorporated into the assessment of rock fragmentation mechanisms and wellbore stability, enabling the co-optimization of drilling efficiency and wellbore safety.

### 4.5. Limitations

While this study provides systematic insights into the mechanical response of granite under in situ HTHP true triaxial conditions, certain limitations should be noted. The experiments were conducted on an outcrop analog rock selected for its similarity to the target reservoir core, meaning the direct in situ microstructural and diagenetic history of the deep formation is not replicated. The tested conditions, though representative of a significant depth range, are bounded by maximum values of 200 °C and 200 MPa. Behavior under even more extreme conditions pertinent to ultra-deep reservoirs remains an open question. Furthermore, this work focused on the short-term mechanical response; the time-dependent behavior (e.g., creep) under such coupled HTHP and true triaxial stress states was not investigated.

### 4.6. Directions for Further Work

The findings and limitations of this study point to several valuable directions for future research. Extending the experimental database to higher temperature and pressure regimes, and incorporating actual downhole cores when possible, would enhance direct applicability. Investigating the time-dependent deformation and failure laws under sustained HTHP true triaxial stress is crucial for long-term engineering assessments. Employing integrated multi-physics monitoring (e.g., acoustic emission, digital image correlation) during testing could provide a more dynamic and comprehensive understanding of the damage evolution process. Finally, the experimental data generated here can serve as a critical benchmark for developing or refining constitutive models that explicitly account for the coupled effects of real-time high temperature and a true triaxial stress state.

## 5. Conclusions

Based on the in situ temperature and pressure conditions at depths of 4000 m, 5000 m, and 5700 m within the Bozhong 19-6 gas field reservoir, this study systematically investigated the evolution of granite mechanical properties under complex thermo-mechanical coupling. High-temperature and high-pressure true triaxial compression tests were conducted to examine the effects of temperature, σ_2_, and σ_3_ on the mechanical properties and fracture morphology. The main conclusions are summarized as follows:

(1) Effect of Temperature: An increase in temperature (25–200 °C) leads to a deterioration of the mechanical properties and an enhancement of the ductility of granite. Specifically, elevated temperature significantly reduces the peak strength and elastic modulus of the rock, while increasing the peak strain. The failure mode transforms from a brittle fracture with distinct macroscopic cracks to a more distributed, damage-dominated failure with more complex fracture networks.
(2) Effect of σ_2_: An increase in σ_2_ significantly enhances the brittleness of granite. This is manifested by a corresponding increase in peak strength and elastic modulus, a decrease in peak strain, and a shift in the failure mode from tension–shear composite failure to shear failure, with the fracture morphology tending to become simpler.
(3) Effect of σ_3_: An increase in σ_3_ results in higher peak stress and peak strain, but a reduced elastic modulus, and a gradual shift in crack propagation from a tensile mode to a shear mode. This effectively inhibits the formation of a single dominant fracture surface, leading to increased fracture complexity.

## Figures and Tables

**Table 1:** Mineral composition of Tai-2 granodiorite and the reservoir rock (unit: %).

Rock Type	Quartz	K-Feldspar	Plagioclase	Chlorite	Biotite	Apatite	Titanite	Anthophyllite	Others
Tai-2	45.5	0.11	31.32	0.35	11.87	0.31	0.2	5.82	4.52
Reservoir Rock	23.1	6.66	33.4	7.72	17.4	0.03	0	0.83	10.86

**Table 2:** Physical and mechanical properties of Tai-2 granodiorite and the reservoir rock.

Rock Type	P-Wave Velocity (m/s)	Uniaxial Compressive Strength (MPa)	Elastic Modulus (GPa)	Poisson’s Ratio
Tai-2	4643	122.39	14.85	0.287
Reservoir Rock	4786	122.43	17.97	0.262

**Table 3:** Actual temperature and pressure conditions at different depths in the study area.

Depth (m)	Temperature (°C)	σ_3_ (MPa)	σ_2_ (MPa)
4000	150	64	80
5000	180	80	100
5700	200	96	120

**Table 4:** Scheme for high-temperature and high-pressure true triaxial tests.

Depth (m)	Temperature and Stress Conditions			Investigated Factor		
	Temperature (°C)	σ_3_ (MPa)	σ_2_ (MPa)	Temperature (°C)	σ_3_ (MPa)	σ_2_ (MPa)
4000	25	64	80	√		
	50	64	80	√		
	100	64	80	√		
	150	64	80	√		√
	150	64	64			√
	200	64	80	√		
	150	64	120		√	√
5000	25	80	100	√		
	50	80	100	√		
	100	80	100	√		
	150	80	100	√		
	180	80	100	√		√
	180	80	80			√
	200	80	100	√		
	180	80	160			√
	150	80	120		√	
5700	25	96	120	√		
	50	96	120	√		
	100	96	120	√		
	150	96	120	√	√	
	200	96	120	√		√
	200	96	96			√
	200	96	200			√

**Table 5:** Summary of mechanical parameters from HTHP true triaxial tests.

Temperature (°C)	σ_3_ (MPa)	σ_2_ (MPa)	σ_c_ (MPa)	ε (MPa)	E (GPa)	μ
25	64	80	601.32	1.20	58.37	0.31
50	64	80	565.96	1.25	55.75	0.35
100	64	80	543.43	1.34	45.70	0.26
150	64	80	540.46	1.41	43.54	0.34
150	64	64	522.39	1.34	44.95	0.31
200	64	80	496.72	1.43	35.33	0.26
150	64	120	624.97	1.46	61.51	0.35
25	80	100	695.00	1.38	58.43	0.30
50	80	100	684.87	1.41	57.42	0.32
100	80	100	675.97	1.46	61.81	0.36
150	80	100	660.77	1.51	58.99	0.38
180	80	100	628.38	1.50	52.84	0.35
180	80	80	550.49	1.55	40.57	0.32
200	80	100	609.09	1.62	49.63	0.32
180	80	160	726.93	1.32	59.40	0.29
150	80	120	694.13	1.42	53.95	0.11
25	96	120	766.33	1.59	69.51	0.22
50	96	120	753.23	1.60	48.20	0.19
100	96	120	736.30	1.61	57.21	0.26
150	96	120	699.45	1.63	48.86	0.30
200	96	120	676.23	1.65	49.51	0.18
200	96	96	652.45	1.67	42.98	0.23
200	96	200	808.52	1.25	74.84	0.16

## Data Availability

The original contributions presented in this study are included in the article/Supplementary Materials. Further inquiries can be directed to the corresponding author.

## Supplementary Materials

The following supporting information can be downloaded at: https://www.mdpi.com/article/10.3390/ma19071396/s1, Figure S1. The non-metallic ultrasonic testing analyzer. Table S1. Measured P-wave and S-wave velocities of the granite specimens.
