# Supplementary material for: Mechanical Response and Failure Characteristics of Granite Under In Situ High-Temperature and High-Pressure True Triaxial Conditions
Source: Materials (Basel). 2026 Mar 31;19(7):1396. doi: 10.3390/ma19071396 (PMC13074230; doi:10.3390/ma19071396)
Supplement: Supplementary file 1 [file materials-19-01396-s001.zip › materials-4206667-supplementary.pdf]

Supplementary Data for Ultrasonic Testing

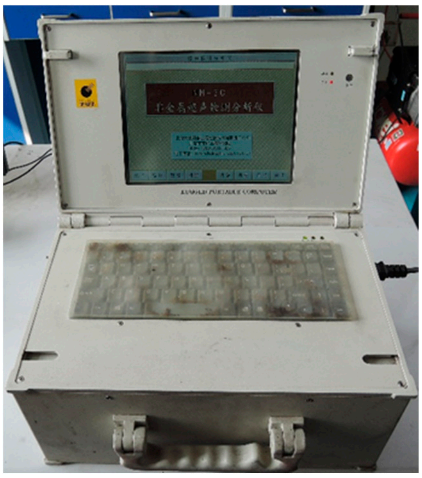

Figure S1. The non-metallic ultrasonic testing analyzer.

Table S1. Measured P-wave and S-wave velocities of the granite specimens.

Specimen ID	P-wave Velocity (m/s)	S-wave Velocity (m/s)	Accepted or not
S-01	4549	2819	√
S-02	4625	2769	√
S-03	4512	2728	√
S-04	4504	2621	√
S-05	4509	2650	√
S-06	4660	2707	√
S-07	4130	2460	×
S-08	4594	2664	√
S-09	4570	2814	√
S-10	4631	2703	√
S-11	4643	2762	√
S-12	4695	2815	√
S-13	4720	2800	√
S-14	4714	2664	√
S-15	4258	2497	×
S-16	4582	2641	√
S-17	4216	2398	×
S-18	4648	2641	√
S-19	4673	2773	√
S-20	4702	2663	√
S-21	4512	2683	√
S-22	4674	2734	√
S-23	4501	2706	√

S-24	4516	2806	√
S-25	4480	2810	√
S-26	4057	2315	×
S-27	4612	2649	√
S-28	4660	2800	√
S-29	4143	2506	×
S-30	4688	2747	√
